# SARS-CoV-2 Seroepidemiology and Antibody Levels in Children during BA.5 Predominance Period

**DOI:** 10.3390/diagnostics14101039

**Published:** 2024-05-17

**Authors:** Filippos Filippatos, Elizabeth-Barbara Tatsi, Maria-Myrto Dourdouna, Emmanouil Zoumakis, Alexandra Margeli, Vasiliki Syriopoulou, Athanasios Michos

**Affiliations:** 1 First Department of Pediatrics, Medical School, National and Kapodistrian University of Athens, “Aghia Sophia” Children’s Hospital, 11527 Athens, Greece; filippat@med.uoa.gr (F.F.); etatsi@med.uoa.gr (E.-B.T.); myrtodour@med.uoa.gr (M.-M.D.); mzoumak@med.uoa.gr (E.Z.); amichos@med.uoa.gr (A.M.); 2 Department of Clinical Biochemistry, “Aghia Sophia” Children’s Hospital, 11527 Athens, Greece; a.margeli@paidon-agiasofia.gr

**Keywords:** SARS-CoV-2, COVID-19, children, BA.5 omicron variant, seroepidemiology

## Abstract

This is a SARS-CoV-2 seroepidemiological study in a pediatric population (0–16 years) during the BA.5 Omicron predominance period in the Athens metropolitan area. Serum samples were tested for SARS-CoV-2 nucleocapsid antibodies (Abs-N), representing natural infection during three periods of BA.5 predominance: 1 May 2022–31 August 2022 (period A), 1 September 2022–31 December 2022 (period B), and July 2023 (period C). Εpidemiological data were also collected. Additionally, in period C, Abs-N-seronegative samples were tested for SARS-CoV-2 spike antibodies (Abs-S). A total of 878 children were tested (males: 52.6%), with a median age (IQR) of 96 (36–156) months; the number of cases of seropositivity during the three periods were as follows: A: 292/417 (70%), B: 288/356 (80.9%), and C: 89/105 (84.8%), with *p* < 0.001. SARS-CoV-2 seropositivity increased from period A to C for children 0–1 year (*p* = 0.044), >1–4 years (*p* = 0.028), and >6–12 years (*p* = 0.003). Children > 6–12 years had the highest seropositivity rates in all periods (A: 77.3%, B: 91.4%, and C: 95.8%). A significant correlation of monthly median Abs-N titers with monthly seropositivity rates was detected (r_s_: 0.812, *p* = 0.008). During period C, 12/105 (11.4%) Abs-S-seropositive and Abs-N-seronegative samples were detected and total seropositivity was estimated at 96.2% (101/105). The findings of this study indicate a high SARS-CoV-2 exposure rate of children during the BA.5 predominance period and suggest that in future seroepidemiological studies, both antibodies should be tested in Abs-N-seronegative populations.

## 1. Introduction

Severe acute respiratory syndrome coronavirus 2 (SARS-CoV-2) is a highly contagious virus which caused the global coronavirus disease 2019 (COVID-19) pandemic [1]. This virus mainly affects the respiratory system, by binding to a receptor called Angiotensin-converting enzyme 2 (ACE2), which is present in specific type 2 alveolar epithelial cells, leading to severe illness and high rates of mortality worldwide [2].

The rapid evolution of the SARS-CoV-2 virus has resulted in distinct variants which are characterized by increased transmissibility and immune invasion strategies [3]. The number of laboratory-confirmed COVID-19 cases has significantly increased, especially following the emergence of BA.1, BA.2, BA.4, and BA.5 Omicron sublineages [4]. BA.5, which was first identified in April 2022 by the World Health Organization, is the most contagious strain of Omicron and is characterized by increased humoral immunity evasion and decreased vaccine effectiveness, while the presence of certain BA.5 gene mutations result in significant decreases in non-BA.5 Omicron antibody-neutralizing activity [5]. Therefore, the impact of BA.5 on SARS-CoV-2 seropositivity compared to other variants of concern is important.

SARS-CoV-2 can be transmitted through household, educational, and community settings by children of any age [6]. Transmission may occur from both symptomatic and asymptomatic children [7]. The majority of pediatric cases were caused by exposure in the household, with an adult typically as the index subject, especially during increased transmissibility variants predominance periods [8]. There have also been reports of health care-associated outbreaks and cases of transmission among students and between students and educational personnel in the school setting, particularly among unvaccinated individuals [7,8,9]. However, transmission by children and adolescents in the school context was more common when strict multiple public health measures were lifted and during the predominance period of more contagious SARS-CoV-2 variants of concern [10].

SARS-CoV-2 confirmed cases in children younger than 18 years old have exceeded 16 million, according to the American Academy of Pediatrics [11]. Although viral transmission rates have been documented in various activities, including educational settings, children are most typically infected by close contact with an infected family member within the same household [12].

Conventional approaches to acute disease surveillance fail to encompass the exact burden of COVID-19 due to factors such as asymptomatic, undetected or unreported cases, especially in pediatric populations and especially after Omicron emergence [11]. Early studies in 2020 support that the SARS-CoV-2 asymptomatic infection ratio in children ranges between 15 and 40% [13]. A study from the United States in 2022 showed that SARS-CoV-2 asymptomatic infections in children during 2020 and 2021 reached 65% [14].

Despite the predominance of different SARS-CoV-2 sublineages, antibody detection remains the most accurate test to confirm a previous SARS-CoV-2 infection. Serologic tests have limited utility for acute SARS-CoV-2 infection diagnosis [15]. Antibody responses comprise the initial production of immunoglobulin M (IgM), which is subsequently followed by an increased circulation of IgA and IgG [16]. The presence of IgG antibodies is typically detectable 16–20 days following infection in the majority of patients [17]. A comprehensive analysis of 38 studies examining the accuracy of serologic testing in COVID-19 patients based on the duration of symptoms revealed that IgM antibodies were identified in 23% of cases within one week, 58% within two weeks, and 75% within three weeks. Similarly, IgG antibodies were detected in 30%, 66%, and 88% of cases, respectively [18]. IgG against the SARS-CoV-2 nucleocapsid protein are produced only after natural infection, while IgG against the SARS-CoV-2 spike protein are produced both after natural infection and vaccination, since the SARS-CoV-2 spike protein is the major antigenic target of currently available SARS-CoV-2 vaccines [19]. Seroprevalence studies based on SARS-CoV-2 natural infection antibodies help determine a more accurate COVID-19 proportion in pediatric populations and offer valuable public health surveillance evidence [20].

SARS-CoV-2 seropositivity was prospectively investigated in an Athens metropolitan area pediatric population by our research group and the results highlighted the increase in SARS-CoV-2 seropositivity in children from the Wuhan strain (1.4%) to Alpha (17.6%), Delta (29.7%), and pre-BA.5 Omicron predominance periods (48.5%) [21,22]. In these studies, the possible role of sex, age, nationality, and hospitalization in different departments in SARS-CoV-2 natural infection seropositivity and antibody levels was evaluated. The predominance period of the BA.5. Omicron sublineage in Europe, including Greece, was estimated to begin in May 2022 [23].

In the current SARS-CoV-2 seroepidemiological study, we aim to investigate SARS-CoV-2 natural infection seropositivity and antibody levels during distinct BA.5 Omicron predominance periods and the association with epidemiological parameters.

## 2. Materials and Methods

### 2.1. Study Design and Participants

This is an observational, population-based prospective seroepidemiological cohort study (based on the STROBE criteria checklist—Supplementary Table S1) conducted at “Aghia Sophia” Children’s Hospital, the largest (750-bed) pediatric hospital in Greece and also a pediatric COVID-19 reference center for the Athens metropolitan area.

Τhe research timeframe was divided into three periods which corresponded to the predominance of the SARS-CoV-2 BA.5 variant in Greece: period A: 1 May 2022–31 August 2022, period B: 1 September 2022–31 December 2022, and period C: July 2023.

Children aged 0–16 years old who were admitted to the hospital or presented to the emergency department for any reason other than confirmed SARS-CoV-2 infection were included in the study. The exclusion criteria were as follows: 1. children who were admitted to the departments of oncology, bleeding disorders, or β-thalassemia because of possible interference with antibody detection due to immunocompromised conditions or transfusions and 2. children readmitted to the hospital on multiple occasions during the study period who had positive antibody test results, in which only the initial positive result was included in the analysis.

Demographic data on sex, age, nationality (Greek or non-Greek), hospitalization status, and hospital department [pediatric departments, emergency department, neonatal intensive care unit (NICU), pediatric intensive care unit (PICU), and surgical departments (surgery, orthopedics, otorhinolaryngology, ophthalmology, urology, cardiothoracic surgery, neurosurgery, and plastic surgery departments)] were also prospectively collected from the hospital medical laboratory analysis system and medical history documents and were included in analysis. Children of all ages were involved in the study and were classified as neonates and infants (0–1 year), toddlers (>1–4 years), pre-school-age children (>4–6 years), school-age children (>6–12 years), and adolescents (>12–16). Children included in period C were also asked for their COVID-19 immunization status. During the study period, no follow-up antibody testing was performed, and the current SARS-CoV-2 study’s seroepidemiological results were based on a single, random antibody measurement.

To prospectively evaluate SARS-CoV-2 infection seropositivity and antibody levels, approximately 100 leftover sera per month were randomly collected from the Department of Biochemistry of “Aghia Sophia’’ Children’s Hospital. Serum samples were collected and stored at −70 °C until laboratory analysis. Serum sample laboratory processes were performed anonymously using a unique individual identification code which was given to the patients upon hospital admission.

### 2.2. SARS-CoV-2 Antibody Detection

All serum samples were analyzed for total (IgM, IgA, and IgG) SARS-CoV-2 nucleocapsid protein antibodies (Abs-N) using Elecsys^®^ Anti-SARS-CoV-2 (Roche Diagnostics, Basel, Switzerland) reagent on a Cobas e 411 immunoassay analyzer according to the manufacturer’s instructions. Values of ≥1 cut-off index (COI) were considered positive.

Serum samples collected in the last period (July 2023) which were negative for Abs-N were also analyzed for SARS-CoV-2 spike protein antibodies (Abs-S; IgM, IgA, and IgG). Abs-S was detected using Elecsys^®^ Anti-SARS-CoV-2 S (Roche Diagnostics, Basel, Switzerland) reagent on a Cobas e 411 immunoassay analyzer according to the manufacturer’s instructions. Antibody values ≥ 0.8 U/mL were characterized as positive.

The principle of electrochemiluminescence immunoassay (ECLIA) for antibody detection is based on a double SARS-CoV-2 specific antigen (nucleocapsid or spike) sandwich enzyme-linked immunosorbent assay (ELISA). The ECLIA method is a highly efficient approach, with an estimated sensitivity of 99.5% (14 days after symptoms appear) and a specificity of 99.8%. According to a study by Riester et al. which recruited 191 pediatric samples, Elecsys^®^ Anti-SARS-CoV-2 specificity is estimated as >99.9% in children [24].

### 2.3. Statistical Analysis

Statistical analysis was performed using Statistical Package for the Social Sciences SPSS version 25.0 (IBM Corp., Armonk, NY, USA, Released 2017). Graphs were created using GraphPad Prism 10 (San Diego, CA, USA). Absolute and relative frequencies (%) were used for the description of qualitative variables (sex, age group, nationality, hospitalization status, and hospital department). The assumption of normality was evaluated with kurtosis and skewness and verified with Kolmogorov–Smirnov and Shapiro–Wilk tests. The median and interquartile range (IQR) were applied for quantitative variables after the assumption of normality was evaluated. X2 tests were applied to assess distinctions among qualitative variables. Differences between qualitative and quantitative variables involved the application of two nonparametric tests: the Mann–Whitney test (qualitative with 2 categories) and the Kruskal–Wallis test (qualitative with >2 categories). The effect size for the Chi-square test was estimated with Cramer’s V. The effect size for the Mann–Whitney test was estimated by r^2^ = Z^2^/*n*, where r is the effect size, Z is the standardized test statistic, and *n* is the total number of observations. The effect size for the Kruskal–Wallis test was the eta squared based on the H-statistic: eta2[H] = (H − k + 1)/(*n* − k), where H is the value obtained in the Kruskal–Wallis test, k is the number of groups, and *n* is the total number of observations. Correlations between quantitative variables were assessed by using the Spearman r correlation coefficient. A multiple linear regression model was performed in order to estimate the SARS-CoV-2-seropositive antibody titers in children (quantitative dependent variable) taking into account the simultaneous interaction of study period, age, sex, nationality, and hospitalization (independent variables), and a 95% confidence interval was used. Statistical significance was set at a *p* < 0.05 level.

### 2.4. Ethical Approval

The study protocol was in accordance with the 1964 Declaration of Helsinki and was approved by the scientific and bioethics committee of “Aghia Sophia” Children’s Hospital (No. 25609). Written informed consent for participation in this study was obtained from the children’s parents.

## 3. Results

### 3.1. Study Population

Serum samples from 878 children with a median age of 96 (IQR = 36–156) months were included in this study. Males represented 462/878 (52.6%). Dividing the population into age groups, 140 (15.9%) were 0–1 year old, 160 (18.2%) were >1–4 years old, 77 (8.8%) were >4–6 years old, 270 (30.8%) were >6–12 years old, and 231 (26.3%) were >12–16 years old. In the study population, 671/878 (76.4%) were of Greek nationality and 617/878 (70.3%) were hospitalized. The majority of children [477/878 (54.3%)] were hospitalized in the pediatric departments of the hospital. The number of serum samples tested in each study period were 417/878 (47.5%) in period A, 356/878 (40.5%) in period B, and 105/878 (12.0%) in period C.

The serums were tested for SARS-CoV-2 Abs-N and a total of 669/878 (76.2%) SARS-CoV-2-seropositive children were detected, having a median age of 108 months (IQR = 36–156 months). Among the 669 seropositive children, 343/669 (51.3%) were males, 502/669 (75%) were of Greek nationality, 459/669 (68.6%) were hospitalized, and 352/669 (52.6%) were hospitalized in the pediatric departments. Detailed demographic and epidemiological characteristics of the study population for each study period are presented in Table 1.

### 3.2. Seropositivity per Period and per Month

**Figure 1:**
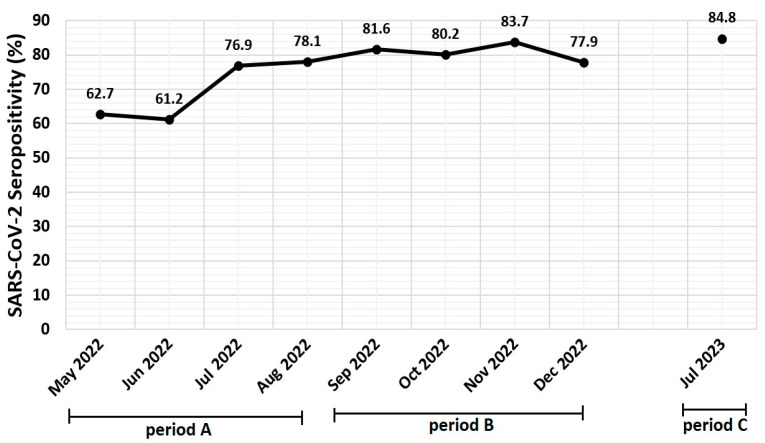
Monthly SARS-CoV-2 seropositivity rates according to antibody detection against SARS-CoV-2 nucleocapsid protein in study population (*n* = 878) (period A: 1 May 2022–31 August 2022, period B: 1 September 2022–31 December 2022, and period C: July 2023).

SARS-CoV-2 seropositivity significantly increased from 292/417 (70.0%) in period A to 288/356 (80.9%) in period B and 89/105 (84.8%) in period C (*p* < 0.001) (Table 1). SARS-CoV-2 seropositivity during each study month also varied significantly (*p* < 0.001) and is presented in Figure 1. The highest seropositivity rate of the whole study period was detected in July 2023 (84.8%; 89/105) and the lowest was detected in June 2022 (61.2%; 60/98) (Figure 1). No statistically significant correlations were identified between SARS-CoV-2 monthly seropositivity rates and newly diagnosed COVID-19 confirmed cases per month in the pediatric (r_s_: −0.209, *p*: 0.589) or total (r_s_: 0.092, *p* = 0.814) population of Greece, respectively [data from European Centre for Disease Prevention and Control (ECDC)] [16].

Twelve out of sixteen (75.0%) SARS-CoV-2 Abs-N-seronegative samples from non-immunized children against COVID-19 (based on medical history data) from period C were positive for SARS-CoV-2 Abs-S. Hence, these SARS-CoV-2 Abs-S-positive samples were added to the seropositivity rate (12/105, 11.4%) and total seropositivity was estimated at 96.2% (101/105).

### 3.3. Association of SARS-CoV-2 Seropositivity with Demographic and Epidemiological Data

No significant differences were detected between seropositive males and females (*p*-value = 0.152, Table 1). SARS-CoV-2 seropositivity rates significantly increased from periods A to C for both sexes (*p* for males = 0.028 and females = 0.002). Females had higher seropositivity rates than males in period C [(92%, 46/50) vs. (78.2%, 43/55), respectively, *p* = 0.049] (Table 1), while no significant differences were detected in period A (*p* = 0.411) and period B (*p* = 0.38) (Table 1). Seropositivity per month varied significantly for females (*p* < 0.001) but not for males (*p* = 0.072). The highest SARS-CoV-2 monthly seropositivity rates were detected in November 2022 for males [44/52 (84.6%)] and in August 2022 for females [48/55 (87.3%)]. The lowest SARS-CoV-2 monthly seropositivity rates were detected in July 2022 for males [30/50 (60%)] and in May 2022 for females [27/48 (56.3%)]. In August 2022, SARS-CoV-2 seropositivity rates between sexes varied significantly [males: 27/41 (65.9%) vs. females: 48/55 (87.3%), *p* = 0.023]. No other significant differences in SARS-CoV-2 monthly seropositivity rates between sexes were detected. SARS-CoV-2 Abs-S seropositivity in period C was estimated at 92.7% (51/55) for males and 100% (50/50) for females (*p* = 0.118).

No differences were detected between seropositive Greek and non-Greek children included in this study (*p* = 0.083, Table 1) in different study periods (A: *p* = 0.24, B: *p* = 0.099, and C: *p* = 0.724; Table 1). Seropositivity in Greek children was significantly increased from periods A to C (*p* = 0.001), but not for non-Greek children (*p* = 0.102) (Table 1). Seropositivity per month varied significantly for Greek children (*p* = 0.001) but not for non-Greek children (*p* = 0.092). The highest SARS-CoV-2 monthly seropositivity rates were detected in July 2023 for Greek children [65/76 (85.5%)] and in September 2022 for non-Greek children [21/22 (95.5%)]. The lowest SARS-CoV-2 monthly seropositivity rates were detected in June 2022 for Greek children [46/79 (58.2%)] and in May 2022 for non-Greek children [14/23 (60.9%)]. SARS-CoV-2 Abs-S seropositivity in period C was estimated at 98.7% (75/76) for Greek children and 89.7% (26/29) for non-Greek children (*p* = 0.304).

SARS-CoV-2 seropositivity rates significantly increased from periods A to C for both hospitalized (*p* = 0.002) and non-hospitalized children (*p* = 0.023). Non-hospitalized children had significantly higher SARS-CoV-2 seropositivity rates compared to hospitalized children (80.5%, 210/261 vs. 74.4%, 459/617; *p* = 0.05) (Table 1). Monthly SARS-CoV-2 seropositivity rates differed significantly in hospitalized children (*p* < 0.001) but not in non-hospitalized children (*p* = 0.25). The highest SARS-CoV-2 monthly seropositivity rates were detected in July 2023 for hospitalized children [68/79 (86.1%)] and in December 2022 for non-hospitalized children [8/9 (88.9%)]. The lowest SARS-CoV-2 monthly seropositivity rates were detected in June 2022 for hospitalized children [35/64 (54.7%)] and in May 2022 for non-hospitalized children [21/29 (72.4%)]. SARS-CoV-2 Abs-S seropositivity in period C was estimated at 89.9% (71/79) for hospitalized children and 88.5% (23/26) for non-hospitalized children (*p* = 0.57).

SARS-CoV-2 seropositivity rates significantly increased from periods A to C in general pediatric departments (*p* = 0.001). No significant differences were reported in SARS-CoV-2 seropositivity among different hospital departments (*p* = 0.177, Table 1). Monthly SARS-CoV-2 seropositivity rates differed significantly in general pediatric departments (*p* = 0.001). SARS-CoV-2 Abs-S seropositivity in period C was estimated at 87.9% (58/66) in pediatrics departments, 100% (2/2) in the NICU, 100% (3/3) in the PICU, 100% (8/8) in surgical departments, and 0% in cardiology/neurology departments, respectively (*p* = 0.659).

### 3.4. Seropositivity in Different Age Groups

**Figure 2:**
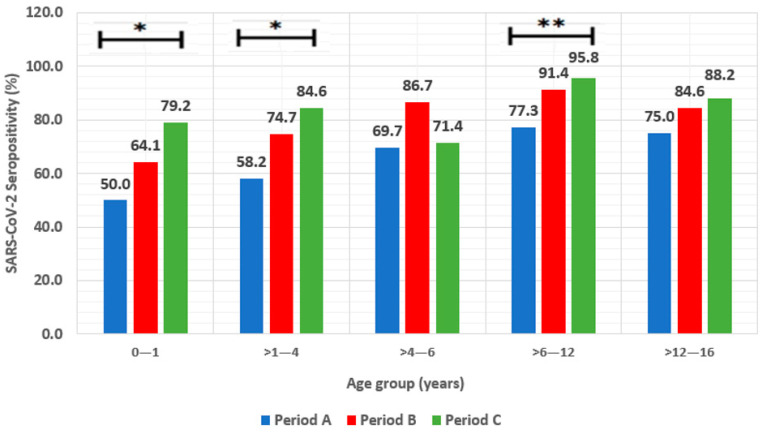
SARS-CoV-2 seropositivity rates per study period (period A: 1 May 2022–31 August 2022, period B: 1 September 2022–31 December 2022, and period C: July 2023) for neonates–infants (0–1 year), toddlers (>1–4 years), pre-school children (>4–6 years), school-age children (>6–12 years), and adolescents (>12–16 years). *: *p* < 0.05; **: *p* < 0.01.

SARS-CoV-2 seropositivity per age group significantly varied in period A (*p* < 0.001) and period B (*p* < 0.001), but no differences were detected in period C (*p* = 0.293) (Figure 2). SARS-CoV-2 seropositivity rates progressively increased from period A to C for all age groups, except for children >4–6 years (*p* = 0.249, Table 1) and >12–16 years (*p* = 0.158, Table 1). Children > 6–12 years had the highest seropositivity rates during the whole study period (84.4%, 228/270) and per period [A: 109/141 (77.3%), B: 96/105 (91.4%), and C: 23/24 (95.8%); *p* = 0.003]. Among age groups, the lowest SARS-CoV-2 seropositivity rates were detected in 0–1-year-old children for the three study periods (61.4%, 86/140) (Figure 2).

In period A, SARS-CoV-2 seropositivity rates per age group varied significantly in May 2022 [0–1 year: 5/16 (31.3%) vs. >1–4 years: 13/22 (59.1%) vs. >4–6 years: 2/3 (66.7%) vs. >6–12 years: 16/21 (76.2%) vs. >12–16 years: 28/40 (70%); *p* = 0.05]. In period B, SARS-CoV-2 seropositivity rates per age group also varied significantly in September 2022 [0–1 year: 8/15 (53.3%) vs. >1–4 years: 15/21 (71.4%) vs. >4–6 years: 8/9 (88.9%) vs. >6–12 years: 25/27 (92.6%) vs. >12–16 years: 24/26 (92.3%); *p* = 0.008].

Total SARS-CoV-2 seropositivity (Abs-S and Abs-N) in period C was estimated at 91.7% (22/24) in children of 0–1 year, 96.2% (25/26) in >1–4 years, 78.6% (11/14) in >4–6 years, 100% (24/24) in >6–12 years, and 100% (17/17) in >12–16 years, respectively (*p* = 0.262).

### 3.5. Association of SARS-CoV-2 Abs-N with Demographic and Epidemiological Data

**Figure 3:**
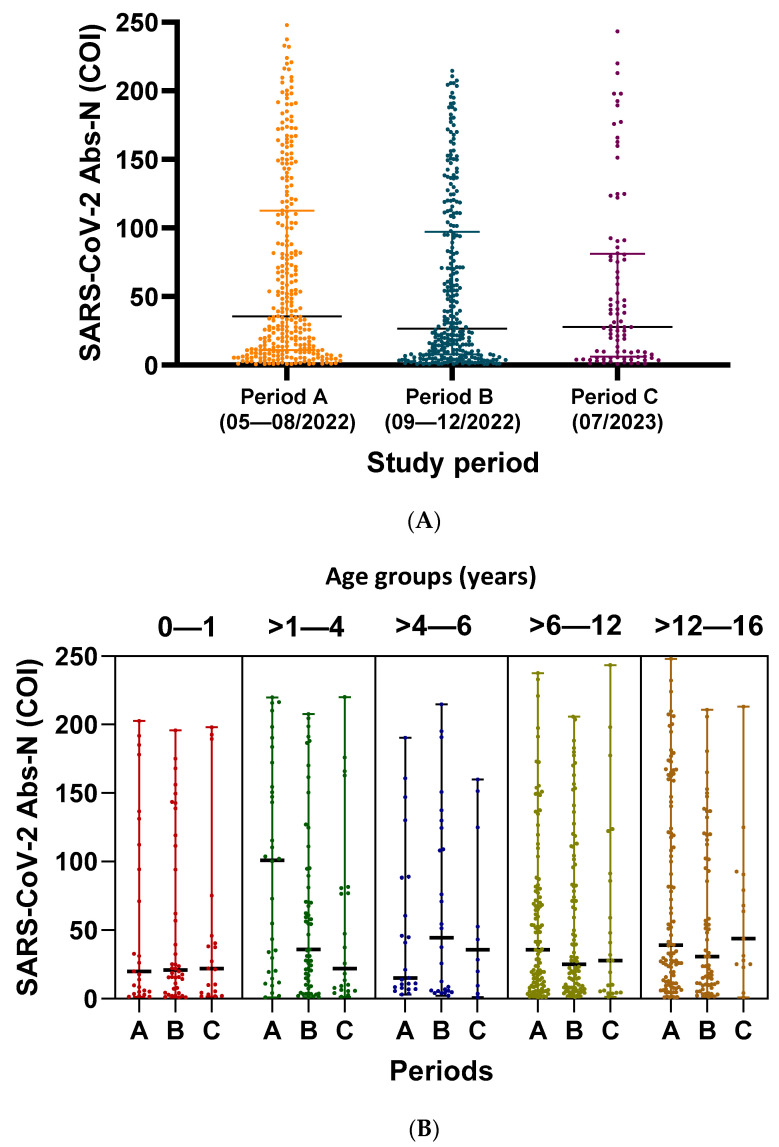
Median values of SARS-CoV-2 Abs-N per study period (Panel (**A**)) and per age group (0–1, >1–4, >4–6, >6–12, and >12–16 years) (Panel (**B**)) of the 669 SARS-CoV-2-seropositive children from period A (1 May 2022–31 August 2022), period B (1 September 2022–31 December 2022), and period C (July 2023). Bold horizontal lines represent median antibody titer values and non-bold horizontal lines represent interquartile range (IQR) values. COI, cut-off index; Abs-N: antibodies for SARS-CoV-2 nucleocapsid protein.

The median value of SARS-CoV-2 Abs-N of the 669 SARS-CoV-2-seropositive children was 31.1 COI (IQR: 9.1–102.5 COI) and did not significantly vary among the three study periods [A: 35.52 COI (IQR: 11.04–112.7 COI) vs. B: 26.48 COI (IQR: 7.87–97.28 COI) vs. C: 27.91 COI (IQR: 6.14–81.12 COI), *p* = 0.119] (Figure 3A), but varied significantly per month (*p* = 0.003). The median value of SARS-CoV-2 Abs-N per month significantly correlated with SARS-CoV-2 monthly seropositivity rates (r_s_: 0.812, *p* = 0.008).

The median values of SARS-CoV-2 Abs-N per age group were estimated at 20.63 COI (IQR: 4.82–94.15 COI) in 0–1-year-old children, 43.5 COI (IQR: 10.05–113.1 COI) in >1–4-year olds, 28.38 COI (IQR: 6.7–108.8 COI) in >4–6-year olds, 29.29 COI (IQR: 8.27–82.47 COI) in >6–12-year olds, and 33.46 COI (IQR: 11.42–119.1 COI) in >12–16-year olds (*p* = 0.08) (Figure 3B). The median values of Abs-N did not correlate significantly with age either during the whole study period (r_s_: 0.064, *p* = 0.098) or in any of the study periods (period A: r_s_: 0.057 and *p* = 0.336, period B: r_s_: 0.023 and *p*: 0.696, and period C: r_s_: 0.125 and *p* = 0.243, respectively).

Non-Greek children had significantly higher median values of Abs-N compared to Greek children [39.58 COI (IQR: 10.46–126.5 COI) vs. 27.42 (IQR: 8.4–93.31 COI), *p* = 0.031]. There were significant differences regarding the median values of SARS-CoV-2 Abs-N among different departments of the hospital (*p* = 0.026). The median values of SARS-CoV-2 Abs-N were higher in children who presented to the emergency department compared to children hospitalized in the NICU [41.30 (IQR: 9.08–113.5 COI) vs. 6.07 (IQR: 2.61–48.72 COI), *p* = 0.036] and higher in the surgical departments compared to the NICU [41.50 (IQR: 14.87–110.7 COI) vs. 6.07 (IQR: 2.61–48.72 COI), *p* = 0.017].

No significant differences were detected regarding the median values of SARS-CoV-2 Abs-N per sex [27.22 COI (IQR: 8.11–95.02 COI) in males vs. 35.12 COI (IQR: 10.18–109.88 COI) in females, *p =* 0.266] and per hospitalization status [27.87 COI (IQR: 9.06–94.68 COI) in hospitalized children vs. 41.31 COI (IQR: 9.07–113.5 COI) in non-hospitalized children, *p =* 0.154].

### 3.6. Multiple Linear Regression Model by Study Period

A multiple linear regression analysis was conducted with the independent variables of study period, age, sex, nationality, and hospitalization and with the dependent variable of SARS-CoV-2 natural disease antibody titers in all SARS-CoV-2-seropositive children (*n* = 669). A statistically significant model was revealed (*p* = 0.031, R^2^ = 0.011), with a significant predictor being the nationality parameter (β = 15.073, *p* = 0.009). The results of the multiple linear regression analysis for the entire study are presented in Supplementary Table S2.

## 4. Discussion

In the present study, we investigated SARS-CoV-2 seropositivity and antibody levels during the BA.5 predominance period in Athens in a pediatric population taking into consideration epidemiological parameters. This is a follow-up study from previous investigations starting from the beginning of the pandemic in March 2020, showing a gradual increase in seropositivity rates in our area from 4% to 52.6% [21,22]. A further increase in SARS-CoV-2 Abs-N seropositivity during the BA.5 predominance period from 62.7% in May 2022 to 84.8% in July 2023 was detected. SARS-CoV-2 seropositivity increased for all age groups, for both sexes, regardless of nationality or hospitalization status, and in all different hospital departments, including emergency departments.

The prospective acquisition of routine leftover residual serum samples from the biochemistry department presents a viable approach for assessing SARS-CoV-2 serosurveillance in children and provides the advantage of avoiding additional blood sampling for pediatric patients. Similar previous studies were also based on the testing of routine leftover residual serum samples, avoiding additional blood sampling for pediatric patients, and this method has successfully been implemented in other seroprevalence studies as well [25,26,27,28].

SARS-CoV-2 seropositivity in our area does not substantially differ from reported seroepidemiological data from the USA, Latvia, Thailand, Guinea, and Cameroon, indicating increased SARS-CoV-2 seropositivity during the BA.5 Omicron period [27,29,30,31].

No differences in SARS-CoV-2 seropositivity rates between sexes were detected, which is also the finding of the Greek National Public Health Organization COVID-19 Surveillance System and other similar studies [23,26,27]. According to our previously published studies, SARS-CoV-2 seropositivity rates between sexes also did not significantly differ either during Wuhan (females: 1.4% vs. males: 1.4%), Alpha (females: 14.6% vs. males: 19.7%), Delta (females: 32.8% vs. males: 27.3%), or pre-BA.5 Omicron (females: 47.7% vs. males: 49.2%) predominance periods, respectively [21,22].

In this study, SARS-CoV-2 infection seropositivity increased for all age groups and was higher especially for school-age children (>6–12 years) and adolescents (>12–16 years). Similar findings from the CDC report that children of 5–11 years, from May to October 2022 in the USA, had the highest SARS-CoV-2 natural infection seropositivity rates (93.5%), followed by 12–17-year-old children (93%) [31]. According to our previously published studies, SARS-CoV-2 natural infection seropositivity rates in Children > 6–12 years gradually increased from 0% in Wuhan to 14.4% in Alpha, 27.8% in Delta, and 56.5% in pre-BA.5 Omicron predominance periods [21,22]. Similarly, SARS-CoV-2 seropositivity rates in children > 12–16 years increased from 0% in Wuhan to 16.9% in Alpha, 32.5% in Delta, and 51.8% in pre-BA.5 Omicron predominance periods, respectively [21,22].

A SARS-CoV-2 seropositivity study in Latvia from May 2022 to July 2022 with 173 children showed that children of 12–18 years had the highest seropositivity rates (94.3%) when compared to those of 5–11 years (90.9%) and <5 years (77.8%) [29]. During the same time period in this study, the highest SARS-CoV-2 seropositivity rates were detected in Children > 6–12 years followed by adolescents. Seropositivity rate differences between the two studies could possibly be attributed to different number of participants and age group stratification. These findings may be attributed to the increased exposure of school-age children in the Omicron period and the enhanced transmissibility of the BA.5 subvariant.

According to a recent meta-analysis, SARS-CoV-2 seropositivity is higher in children in Asian and African regions [32]. These countries are also associated with increased migration rates to Greece over the last few years. In the present study, children of non-Greek nationality had similar seropositivity rates, in contrast with the findings from our previously published data in 2021 and 2022 [21,22]. In our previous studies, SARS-CoV-2 seropositivity rates significantly differed between Greek and non-Greek children during Wuhan (Greek: 1% vs. non-Greek: 2.4%) and Alpha (Greek: 14.3% vs. non-Greek: 25.6%) but not during Delta (Greek: 29.3% vs. non-Greek: 31.5%) or pre-BA.5 Omicron (Greek: 48% vs. non-Greek: 49.7%) predominance periods, respectively [21,22]. However, in the current study, children of non-Greek nationality had higher antibody titers compared to Greek children. This finding could possibly indicate increased exposure that leads to repeated infections among non-Greek underprivileged communities within the Athens metropolitan area.

Due to high SARS-CoV-2 seropositivity rates for natural infection (Abs-N) in our area, we decided to perform follow-up SARS-CoV-2 seroprevalence studies every six months with additional Abs-S testing. From the additional testing, it was shown that even with negative Abs-N, a significant percentage of the SARS-CoV-2 unvaccinated population could have positive Abs-S. After detecting both antibodies, it was found that children of 6–16 years had 100% SARS-CoV-2 seropositivity. According to the CDC, these two pediatric age groups are associated with the highest seroprevalence rates (97.1% for school-age children and 98.9% for adolescents, respectively) and this agrees with the findings of our study [31]. In Greece, where approximately half of the total country population has had laboratory-confirmed COVID-19, SARS-CoV-2 vaccination rates with a complete primary series reach 75% in adults and children [33]. Waning humoral immunity several months following SARS-CoV-2 infection [34] supports that SARS-CoV-2 vaccination is of vital importance to offer a surrogate of protection. The findings of this study suggest that in future seroepidemiological studies, both antibodies should be tested in Abs-N-seronegative populations.

In winter 2022 during the BA.5 predominance period, non-hospitalized children were incommensurately affected by COVID-19 compared to hospitalized children, regardless of being in different hospital departments. In contrast, hospitalized children in Greece were found to have higher seropositivity rates during Alpha and Delta periods [21,22]. Those differences could be explained as more children possibly visited the emergency department during Omicron for mild common cold symptoms due to SARS-CoV-2 reinfection but were not detected as SARS-CoV-2-positive upon admission by molecular or rapid antigen testing. This hypothesis could also explain the increased antibody titers of non-hospitalized children compared to hospitalized children also reported in our study. According to the CDC, there was an increasing trend of emergency department visits due to COVID-19 diagnosis during 2023 for both children and adults [35]. Therefore, SARS-CoV-2 seroprevalence studies in different hospital departments, including emergency departments, could provide valuable epidemiological data or possibly guide hospital measures.

The association of SARS-CoV-2 seropositivity and antibody levels with symptom severity is of vital importance. Cohorts of immunocompromised children, including primary or secondary immunodeficiencies such as antibody deficiencies and autoimmune disorders, or those undergoing immunosuppressive medication treatment or impaired T-cell immune responses are usually SARS-CoV-2-seronegative and have a greater incidence of severe COVID-19 [36,37] in comparison to the general pediatric population. Not only seropositivity, but also antibody levels play a significant role in SARS-CoV-2 symptom severity in children. Lower levels of convalescent antibodies targeting the spike and nucleocapsid proteins are indicative of the impact of disease severity on the antibody response. Specifically, children with asymptomatic or mild infections have lower levels of these antibodies compared to those who require hospitalization due to moderate COVID-19 [38,39]. Children with MIS-C have been found to have elevated levels of neutralizing and total antibodies against the spike protein in comparison to children with acute COVID-19 [40].

A limitation of the present study was that Abs-N, representing natural infection, was analyzed for the total population and Abs-S, which could be also vaccine-induced, was additionally analyzed only for the last period of the study. However, children with positive Ab-S were not immunized against COVID-19. The limited number of participants in period C and especially in the age group of >4–6 years could also result in potential bias in the statistical analysis. The use of multiple statistical tests without adjustment for multiple comparisons could increase the risk of type I errors (false positives); therefore, these results should be interpreted with caution.

## 5. Conclusions

The increased SARS-CoV-2 seropositivity rate detected in the current seroepidemiology study illustrates the high exposure of children during the BA.5 predominance period. A key finding of this study is the positive association of SARS-CoV-2 seropositivity with age in the pediatric population, with almost all school-age children and adolescents having been exposed to SARS-CoV-2 during BA.5. Another key finding is that some SARS-CoV-2-unvaccinated Abs-N-seronegative children were Abs-S seropositive, thus having a minimum surrogate of protection against future SARS-CoV-2 reinfection. A future study for SARS-CoV-2 Abs-N kinetics in children with sex- or age-matched SARS-CoV-2 Abs-S controls (control group) could assist in clarifying the difference between Abs-N and Abs-S kinetics during BA.5 predominance in terms of seropositivity rates. Since most children have been exposed to SARS-CoV-2, future studies should probably focus on neutralization assays or T-cell immunity to new SARS-CoV-2 variants to guide public health decisions.

## Figures and Tables

**Table 1:** A comparison of SARS-CoV-2 seropositivity rate (%) for each demographic and epidemiological parameter among the three study periods.

		Study Periods			
	Total *n* (%)	A *n* (%)	B *n* (%)	C *n* (%)	*p*
Seropositive	669/878 (76.2)	292/417 (70.0)	288/356 (80.9)	89/105 (84.8)	**<0.001**
Sex					
Male	343/462 (74.2)	139/204 (68.1)	161/203 (79.3)	43/55 (78.2)	**0.028**
Female	326/416 (78.4)	153/213 (71.8)	127/153 (83.0)	46/50 (92.0)	**0.002**
*p*	0.152	0.411	0.38	**0.049**	
Age group (years)					
0–1	86/140 (61.4)	26/52 (50.0)	41/64 (64.1)	19/24 (79.2)	**0.044**
>1–4	113/160 (70.6)	32/55 (58.2)	59/79 (74.7)	22/26 (84.6)	**0.028**
>4–6	59/77 (76.6)	22/33 (66.6)	26/30 (86.7)	10/14 (71.4)	0.249
>6–12	228/270 (84.4)	109/141 (77.3)	96/105 (91.4)	23/24 (95.8)	**0.003**
>12–16	183/231 (79.2)	102/136 (75.0)	66/78 (84.6)	15/17 (88.2)	0.158
*p*	**<0.001**	**0.001**	**<0.001**	0.293	
Nationality					
Greek	502/671 (74.8)	218/318 (68.6)	219/277 (79.1)	65/76 (85.5)	**0.001**
non-Greek	167/207 (80.7)	74/99 (74.7)	69/79 (87.3)	24/29 (82.8)	0.102
*p*	0.083	0.24	0.099	0.724	
Hospitalized					
Yes	459/617 (74.4)	199/291 (68.4)	192/247 (77.7)	68/79 (86.1)	**0.002**
No	210/261 (80.5)	93/126 (73.8)	96/109 (88.1)	21/26 (80.8)	**0.023**
*p*	**0.05**	0.267	**0.022**	0.514	
Department					
Pediatrics	352/477 (73.8)	145/219 (66.2)	151/192 (78.6)	56/66 (84.8)	**0.001**
NICU	22/29 (75.9)	11/14 (78.6)	9/13 (69.2)	2/2 (100)	0.605
PICU	11/18 (61.1)	5/10 (50.0)	4/5 (80.0)	2/3 (66.7)	0.52
Surgical	51/66 (77.3)	29/36 (80.6)	14/22 (63.6)	8/8 (100)	0.086
Cardiology–Neurology	23/27 (85.2)	9/12 (75.0)	14/15 (93.3)	0/0 (0)	0.183
*p*	0.177	0.234	**0.048**	0.578	

Study periods: period A = 1 May 2022–31 August 2022, period B = 1 September 2022–31 December 2022, and period C = July 2023. Statistically significant differences (*p* < 0.05) are marked in bold.

## Data Availability

All relevant data are published within this paper.

## Supplementary Materials

The following supporting information can be downloaded at: https://www.mdpi.com/article/10.3390/diagnostics14101039/s1, Table S1: STROBE statement—checklist of items that should be included in reports of cohort studies; Table S2: Multiple linear regression model analysis of 669 SARS-CoV-2-seropositive children for whole study period (1 May 2022–31 December 2022 and July 2023), involving SARS-CoV-2 natural infection antibody titers as dependent variable and period, sex, age, nationality, and hospitalization status as independent variables. Statistically significant differences (*p* < 0.05) are marked in bold.
